# Treatment of Scarlet Fever
*On Scarlatina, its Nature and Treatment. By I. Baker Brown, F.R.C.S. Second Edition. Churchill. 1857.


**Published:** 1857-04

**Authors:** 


### THE EPITOME OF SANITARY LITERATURE.

### TREATMENT OF SCARLET FEVER.*

MR. BROWN opens his treatise with a chapter on the mortality of scarlet fever. He shows from the writings of Drs. Copland, Gregory, and Richardson, that this epidemic stands, next to typhus, highest in the mortality scale. In the second chapter he dwells on the origin, recurrence, and the contagious character of the disease, and on the influence of age, sex, and season on its development. Passing then to the varieties of scarlet fever, and its sequelæ, he comes to the indications of treatment, as deduced from the pathology, and proposes the use of his favorite remedy, acetic acid. The form in which this remedy is administered is as follows: dilute acetic acid, two drachms; syrup, four drachms; water, two ounces; of which a fourth part is taken every three hours. The patient's room he recommends to be kept at an equable temperature, well ventilated, and not over-heated, as is often the case; he advises that the patient be not smothered in blankets, from the idea that great warmth is needed, although he would avoid all currents of air which may chill the surface and stop the eruption; as the disease, he tells us, is rarely got over when its course is arrested at this stage. Sponging the skin with tepid vinegar and water is another suggestion made by our author, who finds it to administer great comfort to the patient, by allaying the irritation of the skin, which is often most distressing, and producing an agreeable feeling of general warmth over the surface, which induces sleep where no anodyne has the slightest effect. In summing up, Mr. Brown says that the leading principle of his treatment is based on the idea that scarlet fever is a disease of debility, and that depressing measures are all injurious. His book will be read with interest and advantage.

* On Scarlatina, its Nature and Treatment. By I. BAKER BROWN, F.R.C.S. Second Edition. Churchill. 1857.

